# What is your diagnosis?

**DOI:** 10.4274/jtgga.galenos.2019.2019.0007

**Published:** 2019-02-26

**Authors:** Anupama Bahadur, Namrata Bhattacharya, Latika Chawla, Kavita Khoiwal, Prashant Durgapal, Jaya Chaturvedi

**Affiliations:** 1 Department of Obstetrics and Gynecology, All India Institute of Medical Sciences, New Delhi, India; 2 Department of Pathology, All India Institute of Medical Sciences, New Delhi, India

A 42-year-old lady, para 2 with 2 living issues, presented to us with symptoms of continuous bleeding per vaginum for 15 days following two months of amenorrhoea. She had associated left lower abdominal pain. Her vitals were stable and on per abdominal examination a 16-18 weeks size abdominopelvic mass, firm in consistency, irregular, tender with restricted mobility was palpable. On per vaginal examination, the uterus was irregularly enlarged to 16-18 weeks’ size. A 5×5-cm firm, irregular, tender mass was palpated in the left fornix. This mass could not be demarcated separately from the uterus. Cervical motion tenderness was elicited. On ultrasound, an irregularly enlarged 14-16 weeks’ size uterus with multiple myomas was seen, largest 10×9×9 cm at the left cornua, with intramural and sub-serosal component. The left ovary could not be seen separately from the mass. The right ovary was normal in size and location. Urine pregnancy test and serum beta human chorionic gonadotropin were found to be negative. With suspicion of chronic ectopic pregnancy, the patient was planned for a laparotomy. Intraoperatively, around 200 mL hemoperitoneum was found. Uterus was 14-16 weeks’ size enlarged, with multiple fibroids. A large 10×10 cm irregular friable mass with blood clots was observed at the left cornua extending into the left adnexa. The left tube and ovary were adhered to the mass and were not seen separately. Right tube and ovary was normal (Figure 1). In view of multiple fibroids, total abdominal hysterectomy with bilateral salpingo-oophorectomy was performed (Figure 2).

## Answer

Total abdominal hysterectomy with bilateral salpingo-oophorectomy was performed and a sample was sent for histopathologic examination. The histopathology report suggested leiomyosarcoma involving the myometrium and left ovary, with French Federation of Comprehensive Cancer Centers histologic grade 2 (total score: 1+2+1=4). Here, the primary tumour extends beyond the uterus (pT2NxM_not applicable_) and regional lymph nodes cannot be assessed (pN_x_). Postoperative period was uneventful and patient was discharged in a satisfactory condition after 4 days. She was referred to the radiotherapy department for further management. The patient is doing well 6 months post-operatively.

Uterine leiomyosarcoma is a rare uterine malignancy, originating from smooth muscle of the uterine wall. The median age of occurrence of leiomyosarcomas is 43 to 53 years. The incidence of sarcomas in patients undergoing surgery for leiomyomas has been observed to be around 0.23% (1). Leiomyosarcoma is the commonest histopathologic variant of uterine sarcomas (2). The presenting symptoms include heavy menstrual bleeding, pelvic pain or pressure and occasionally an abdominopelvic mass. We report a case of a 42-year-old female who presented with irregular menstrual cycles, pelvic pain, and bleeding per vaginum, and was later diagnosed as having leiomyosarcoma of the uterus.

Ectopic pregnancy is considered to be a great mimic in gynecology (3) and chronic ectopic pregnancy poses a challenge because of its subtle symptoms and wide range of clinical presentation. A rare case report by Sinha et al. (4) describe a case of chorioadenoma destruens mimicking ruptured ectopic pregnancy. A case report by Sakamoto et al. (5) reported gestational choriocarcinoma with uterine serosal metastasis mimicking ruptured ectopic pregnancy. Ogu et al. (6) reported a case of submucous uterine fibroid mimicking ruptured ectopic gestation. Primary ovarian choriocarcinoma mimicking ectopic pregnancy has been reported by Heo et al. (7). In a study by Leitao et al. (8), the percentage of ovarian metastasis secondary to uterine leiomyosarcoma was 5.4%. However, the incidence of ovarian metastasis in a case of uterine leiomyosarcoma in India is not well documented. The most common mode of spread in leiomyosarcoma is hematogenous with lymphatic spread being rare, and hence lymph node dissection may be omitted in leiomyosarcoma where its therapeutic and diagnostic value is questionable (9).

## Figures and Tables

**Figure 1:**
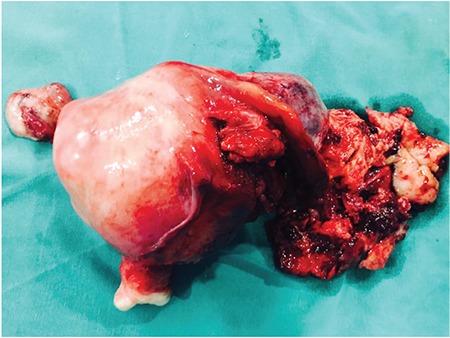
Uterus with bilateral tubes and ovaries showing leiomyosarcoma involving the right ovary, mimicking chronic ectopic pregnancy

**Figure 2:**
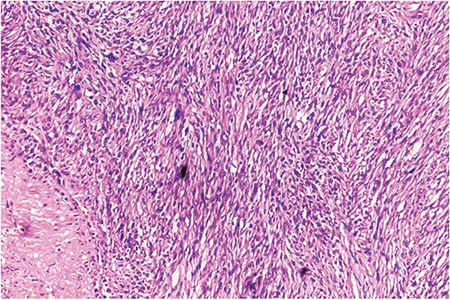
Showing fascicles of spindle cells with necrosis, marked nuclear pleomorphism and increased mitoses showing features of leiomyosarcoma
